# An Efficient Data-Gathering Routing Protocol for Underwater Wireless Sensor Networks

**DOI:** 10.3390/s151129149

**Published:** 2015-11-17

**Authors:** Nadeem Javaid, Naveed Ilyas, Ashfaq Ahmad, Nabil Alrajeh, Umar Qasim, Zahoor Ali Khan, Tayyaba Liaqat, Majid Iqbal Khan

**Affiliations:** 1 COMSATS Institute of Information Technology, Park Road, Islamabad 44000, Pakistan; E-Mails: naveedilyas.ajk@gmail.com (N.I.); ashfaqcomsats@gmail.com (A.A.); majid_iqbal@comsats.edu.pk (M.I.K.); 2 College of Applied Medical Sciences, Department of Biomedical Technology, King Saud University, Riyadh 11633, Saudi Arabia; E-Mail: nabil@ksu.edu.sa; 3 Cameron Library, University of Alberta, Edmonton, AB T6G 2J8, Canada; E-Mail: umar.qasim@ualberta.ca; 4 Internetworking Program, FE, Dalhousie University, Halifax, NS B3J 4R2, Canada; E-Mail: zahoor.khan@dal.ca; 5 Institute of Space Technology, Islamabad Highway, Islamabad 44000, Pakistan; E-Mail: tayyaba.bet091@gmail.com

**Keywords:** underwater wireless sensor networks, routing protocol, mobile sink, autonomous underwater vehicle, lifetime

## Abstract

Most applications of underwater wireless sensor networks (UWSNs) demand reliable data delivery over a longer period in an efficient and timely manner. However, the harsh and unpredictable underwater environment makes routing more challenging as compared to terrestrial WSNs. Most of the existing schemes deploy mobile sensors or a mobile sink (MS) to maximize data gathering. However, the relatively high deployment cost prevents their usage in most applications. Thus, this paper presents an autonomous underwater vehicle (AUV)-aided efficient data-gathering (AEDG) routing protocol for reliable data delivery in UWSNs. To prolong the network lifetime, AEDG employs an AUV for data collection from gateways and uses a shortest path tree (SPT) algorithm while associating sensor nodes with the gateways. The AEDG protocol also limits the number of associated nodes with the gateway nodes to minimize the network energy consumption and to prevent the gateways from overloading. Moreover, gateways are rotated with the passage of time to balance the energy consumption of the network. To prevent data loss, AEDG allows dynamic data collection at the AUV depending on the limited number of member nodes that are associated with each gateway. We also develop a sub-optimal elliptical trajectory of AUV by using a connected dominating set (CDS) to further facilitate network throughput maximization. The performance of the AEDG is validated via simulations, which demonstrate the effectiveness of AEDG in comparison to two existing UWSN routing protocols in terms of the selected performance metrics.

## 1. Introduction

Recent research has witnessed tremendous research interest in UWSNs because of the wide range of applications. Example applications include oil and gas pipeline monitoring, coastline surveillance, underwater mine detection and oceanographic data collection [1,2]. These networks employ stringent resource-constrained sensors [3] to monitor a phenomena (e.g., the pH level of water) and report it to the sink, which relays it to the surface station [4,5].

Due to the rapid attenuation and high absorption of radio signals in UWSNs, communication via these signals is not a feasible method. Thus, acoustic signals are widely used for such purposes, as these have a relatively low absorption rate. However, due to the featured low bandwidth and high end-to-end delay of acoustic signals, the routing task in UWSNs becomes highly challenging. Another unique characteristic of the aqueous environment is its highly dynamic network topology, which demands frequent information exchange (significant overhead) between the nodes if the network is to operate properly. Limited energy resources further make the life of nodes in demand for longevity. In short, enhanced energy efficiency, network lifetime prolongation, reliability improvement, end-to-end delay minimization and efficient data gathering are always desired while designing a UWSN routing protocol [6,7,8].

For efficient and reliable data collection, most of the existing routing protocols employ a static or mobile sink to the improve network lifetime [5,9,10]. The former badly suffers from a hot-spot problem, in which nearby sensors to the sink die out more quickly due to consistent energy consumption in relaying data of distant nodes. To cope with this problem, some researchers employed mobile nodes. The rationale is that continuously changing the neighbours of a sink will result in balanced energy consumption. To minimize energy consumption, a few schemes are proposed that deploy an mobile sink (MS), which moves closer to sensors for data collection. If the network area is increased, then four possible cases are worth discussing.

(i) Neither the number of nodes is increased, nor is the AUV added: In this case, the nodes communicate at relatively farther distances. Thus, the performance of the network degrades in terms of network lifetime, throughput, end-to-end delay, *etc.*
(ii) The number of nodes is increased, but the AUV is not added: Initially, throughput and network lifetime would increase; however, later on, both will decrease due to interference. The end-to-end delay, in this case, would increase.
(iii) The AUV is added, but the number of nodes is not increased: The network lifetime and throughput would increase, and the end-to-end delay would decrease.
(iv) The number of nodes is increased, and the AUV is added: This case would show hybrid results of Case (ii) and Case (iii).

In this research work, we propose an efficient data-gathering scheme using an autonomous underwater vehicle (AUV). We begin our findings by developing a sub-optimal elliptical AUV trajectory via a connected dominated set (CDS). The semi-major and semi-minor axis of the elliptical path are sub-optimized to enhance data gathering in the harsh underwater environment. In this regard, the Monte Carlo simulation method is used to calculate the sub-optimal value of *β* (defined in Section 4). Next, we precede our findings with the selection of gateway nodes (GNs), which are later on rotated on the basis of residual energy (this rotation balances the network’s energy consumption). Soon after the selection of GNs, member nodes (MNs) are associated with GNs by using the shortest path tree (SPT) algorithm [11]. It is worth mentioning here that the MNs are minimized in number, as a suitable number of MNs assigned with their respective GNs leads not only to low energy consumption, but also to a decreased number of dropped packets. In this way, data are gathered with minimum loss and reduced energy consumption.

The rest of the paper is organized as follows. Section 2 states the literature work and motivation. Section 3 deals with the definitions of AUV trajectories and finding the shortest path for data collection. Section 4 deals with AUV mobility. Section 5 is based on sub-optimal data gathering. Section 6 discusses the proposed AEDG scheme. Section 7 discusses the simulation results. Section 8 concludes the paper along with future research directions. Finally, references are given at the end of the paper.

## 2. Related Work and Motivation

Due to varying underwater conditions, recent data gathering and network layer protocols face many challenges [12,13]. Some of the existing proposed schemes use GNs to collect data from MNs and transmit it to the MS, where nodes with relatively high received signal strength indicator (RSSI) values are selected as GNs [14].

In [15], the authors describe various factors affecting the underwater communication environment. They investigate the characteristics of the underwater acoustic channel and its impact on the data link and network layers. The performance of autonomous underwater robotic systems (AURS) greatly depends on reliable and efficient communications. A short communication range makes the terrestrial electromagnetic and optical communication techniques infeasible in an underwater environment. Thus, acoustic technology is invoked as the most appropriate technology in the harsh and unpredictable conditions of the ocean. Despite their merits, acoustic signals suffer from time-varying multi-path fading, Doppler shift and strong attenuation at high frequencies.

In [16], the authors propose a new mathematical technique to find the distance between nodes in a UWSN. This mathematical scheme relies on the RSSI value. In their work, a hybrid computation technique is introduced, which inverts the transmission loss using the Lambert W function (calculated by the Halley method).

The authors in [17] propose a round-based clustering scheme for data redundancy resolving (RBCDRR). In this research work, the authors propose a cluster-based approach to deal with data redundancy. The cluster heads are selected on the basis of the nodes’ residual energy and relative communication distance from the base station. In order to reduce the data redundancy, data aggregation with Euclidean distance is applied in the cluster. Every time the cluster head changes, new inter-cluster and intra-cluster communication is set up.

The basic ant colony optimization algorithm (ACOA) is a heuristic algorithm that is not only robust, but can also easily be combined with other algorithms. However, convergence at the local solution is its major disadvantage. On the other hand, the artificial fish swarm algorithm (AFSA) quickly converges at the global solution; however, it has lower precision while finding the global solution. Thus, the authors in [18] combine AFSA and ACOA in a self-adaptive manner while searching for an optimal routing path. The proposed ACOA-AFSA routing algorithm possesses the advantages of ACOA and AFSA, such that both the transmission delay and energy consumption of the network are minimized.

In [19], the authors present a routing protocol for UWSNs. This protocol aims to maximize data collection and to overcome the energy hole problem. Initially, the network area is divided into different zones, such that the AUV moves on a pre-defined path for data gathering. Subject to saving scarce energy resources, the authors use sleep and awake mechanisms in the respective zones.

In UWSNs, the major challenges are inefficient data gathering, minimum network lifetime, continuous variation in topology and high energy consumption cost during data transmission. Different routing techniques (in UWSNs) have been proposed to tackle the above-mentioned challenges. These techniques use the static sink, as well as the MS approach. In the latter approach, the AUV traverses the network and collects data from each GN for efficient data gathering and network lifetime maximization. However, these techniques do not devise a criterion to limit the number of associated MNs with their respective GNs, so that the high data loss and increased energy consumption of GNs can be minimized. In the AUV-aided underwater routing protocol (AURP), fixed GNs are used to collect data from the MNs [20]. Therefore, GNs consume high energy in relaying excessive data. The quick energy depletion of GNs leads to short network lifetime and also results in a low data delivery ratio at the sink. In the extended scheme of the AUV-aided energy-efficient routing protocol (AEERP) [14], GNs are rotated according to their residual energy levels [14]. However, no mechanism exists to limit the number of associated MNs with their respective GNs. Thus, the association of a large number of MNs causes high energy consumption and more data loss at the GNs. Therefore, we propose an AUV-aided data-gathering scheme that limits the number of associated MNs to their respective GNs while moving the AUV on a sub-optimal elliptical path.

## 3. System Model

In this section, we briefly discuss our system model. In this regard, we state the assumptions made, the objectives and the setting. After these, we will discuss the network model and the AUV trajectories in the upcoming subsections.

Assumptions: In this paper, all of the assumptions are based on a centralized solution to schedule transmissions, the knowledge of the AUV location, the knowledge of the nodes’ energy levels, *etc.*

- Central power rests with the sink.
- Nodes always have data to send.
- All nodes are position aware.

Objectives: This paper aims at network lifetime prolongation and throughput maximization (data loss minimization).

Settings: These are as follows.

- The network nodes are hierarchical, *i.e.*, nodes are of four types; MNs, GNs, AUV and sink. The MNs and GNs are randomly deployed in the network field; the AUV moves on its defined trajectory; and the sink resides at the water surface.
- MNs gather the information of interest from their surroundings and send the gathered data to their respective GNs within their allocated time slots. The AUV moves on its defined path and gathers data from in-range GNs. Finally, the AUV transmits gathered data to the sink.
- Nodes, while sending data to other nodes, update them with their residual information via a field in the packet header.
- In order to match the changing GNs with the fixed route of AUV, the GNs are selected within the communication range of AUV, and a synchronization mechanism is defined between these, which will be discussed in the network model.

### 3.1. Network Model: Basic Definitions

We consider a UWSN in which the nodes are responsible for gathering the information of interest from the underwater environment and transmitting these sensed data to the GNs, which in turn forward the received data to the AUV. The AUV, which gathers data from all of the GNs, transmits these to the nearby sink. We model the UWSN as a graph G=(V,E), where *V* represents sensor nodes and *E* represents links (edges) between them; ∀(i,j)∈E. The network area is divided into two parts; direct communication area $\left(A_{DC}\right)$ and multi-hop communication area $\left(A_{MC}\right)$. Nodes lying in the first sub-area are selected as GNs based on their residual energy. Later on, these GNs are rotated to balance the network’s energy consumption. Sensor nodes, other than GNs, are called member nodes (MNs). The MNs are associated with GNs on the basis of localized information for the shortest path. Subject to maximization of data gathering while improving the network’s energy efficiency, the details are as follows.

**Figure 1:**
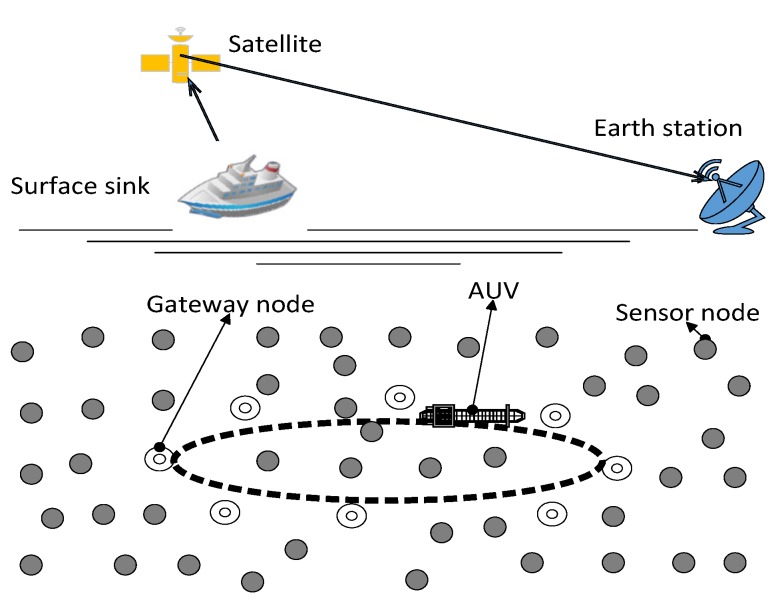
AUV-aided architecture of a UWSN.

In our scenario, *n* number of sensor nodes are randomly deployed over the seabed. Nodes close to the trajectory of AUV are selected as GNs on the basis of the RSSI value, and after that, their role is rotated on the basis of the residual energy of individual nodes. The GNs are rotated at the cost of signalling messages (overhead) exchanged between the AUV and the nodes to be selected as GNs. However, this overhead has no significant impact on the energy efficiency and throughput degradation as compared to the achievements made due to the GNs’ rotation. In order to efficiently communicate within the assigned time slots (to prevent a single point of failure), the AUV needs to be synchronized with the nodes (to be selected as GNs). Within the expected time slot, the AUV listens for the packet (data or control). Upon reception, the AUV compares its current reception time with the expected reception time and calculates a drift value. If $D_{a}$ is the acceptable delay, a drift value is calculated. If the drift value is greater than $D_{a}$, a piggyback mechanism is used for future synchronization. The nodes away from the AUV choose different GNs as their local destinations. AUV moves along the elliptical path to collect data, as shown in Figure 1. We assume that the AUV has unlimited energy and computing resources. Data are relayed to the GNs through the SPT algorithm. Efficient data gathering in UWSNs is a big challenge due to the dynamic and harsh conditions of the ocean, like high propagation delay, packet loss due to multiple transmissions and receptions, high attenuation and multi-path fading. The sub-optimal elliptical path of the AUV may overcome these challenges to some extent.

Note: The GNs are rotated on the basis of residual energy, and the SPT is used to forward the sensed data of the MNs to their respective GNs. Since the SPT takes into consideration the communication distance, it is a feasible choice because the distance is directly related to the energy consumption of the MNs. In other words, our proposed work takes into consideration both residual energy and communication distance. More specifically, residual energy is considered at the time of the GNs rotation, and communication distance is considered at the time of the MNs association with their respective GNs. According to the Monterey-Miami parabolic equation (MMPE) model, distance is directly proportional to path loss. In other words, received signal strength increases as the communication distance between the sender and receiver decreases. Distant communication between nodes leads to a high bit error rate (BER). More specifically, received data with a low signal strength need retransmission(s) to achieve an acceptable level. However, in doing so, surplus energy is consumed. Thus, to avoid this surplus energy consumption, we have considered communication distance at the time of the MNs association with their respective GNs. In this way, the energy of the network is conserved. The rotation of the GNs has a good effect on the network performance in terms of the selected performance metrics. In order to support our claim, we have conducted simulations with and without rotating GNs (refer to the simulation results section). Since this rotation has an effect on the energy consumption of the network, the GNs’ rotation needs to be optimized on the expected energy cost of the bottle neck node. However, this requires much extra work. Thus, for now, we use a non-optimized/heuristic approach for the nodes rotation. 

One may argue that we do not need an acoustic model to know that a larger distance consumes more energy, because by minimizing the hop distance, we may end up transmitting over more hops. However, the overall proposed scenario is not only somehow different, but also more focused on efficient data gathering. The nodes do not directly communicate with the sink; rather, they follow a hierarchical architecture: MNs, GNs, AUV and sink. Here, our objectives are not limited to energy efficiency; rather, these include reliability, as well. Thus, we actually need the acoustic model, whose details are given in the previous paragraph.

In the undirected graph G=(V,E), *V* is further divided into V${}_{CDS}$ and V${}_{non-CDS}$ sets, such that, V${}_{CDS}$⊂V and V${}_{non-CDS}$⊂V. We can define V of graph G as V = V${}_{CDS}$∪ V${}_{non-CDS}$, where V${}_{CDS}$ is a set of CDS nodes and V${}_{non-CDS}$ is a set of non-CDS nodes; moreover, V${}_{CDS}$∩ V${}_{non-CDS}$ = *∅*. Similarly, edges, E, are subdivided into E${}_{CDS}$ and E${}_{non-CDS}$, such that E = E${}_{CDS}$∪ E${}_{non-CDS}$. E${}_{CDS}$ is a set of edges between two CDS nodes. E${}_{non-CDS}$ is a set of edges between non-CDS nodes or CDS and non-CDS nodes. Edges in E${}_{CDS}$ are further divided into E${}_{MST}$ and E${}_{non-MST}$ (MST = minimum spanning tree).

A dominating set (DS) of graph *G* is a subset *D* of *V*, such that every vertex in V\D is adjacent to at least one member vertex of D. A CDS is defined as a subset *D* of *V*, such that any node in *D* can access any other node in *D* by a path that lies entirely within *D*, such that *D* induces a connected sub-graph within *G*.

The CDS is developed by using the extended localized algorithm presented by Dai and Au in [21]. After establishment of CDS, the MST of CDS is constructed. MST is constructed by using the Euclidean distance between nodes *i* and *j*. After the MST, the Hamiltonian circuit (HC) is developed, which is a random trajectory of the AUV. The sub-optimal trajectory for the AUV leads to enhanced network throughput. The criterion for the eligibility of nodes to be included in CDS is shown in the flow chart. CDS formation includes the following steps:

- First of all, check the degree of nodes. After that, consider those nodes that have the highest degree and then the second highest degree. The highest degree nodes are called dominator nodes. The nodes that are adjacent to the highest degree nodes are called dominatee nodes.
- Dominatee nodes that are not further adjacent to any other node are called leaf nodes.
- Dominatee nodes other than the leaf nodes are converted to dominators if that dominatee node has only leaf neighbours.
- The node that is the dominatee of two dominator nodes is also converted to a dominator node.
- The dominator nodes make the CDS in which every node is able to access any other CDS node by a path that exist entirely within the CDS, and every non-CDS node is adjacent to at least one CDS node.

It seems logical that GNs would be (or close to) nodes in CDS (instead of rotating). However, if the GNs are not regularly rotated (on the basis of residual energy), then these will continuously consume relatively high energy that would lead to their earlier death. Afterwards, the far-away nodes will also consume high energy due to distant communication. Thus, the overall network lifetime will be shortened. In order to prolong the network lifetime, the GNs are thus regularly rotated on the basis of residual energy.

Definition 1: A CDS of graph G=(V,E) is a set of nodes V${}_{CDS}$⊂*V*, such that every node *v* belongs to V\D; there is at least one node *u* in V${}_{CDS}$ that dominates v. Moreover, nodes in V${}_{CDS}$ are connected to each other.

Let us consider the example in which nodes are randomly deployed in a network field. For the sake of simplicity, we take 15 sensor nodes to establish the CDS. The criterion for CDS construction in our protocol is discussed above. In this example, the highest degree node is 7, and the second highest degree node is 13. These two nodes are called dominators and, thus, included in the CDS. The second step is to check the dominatee of the dominator for the above-mentioned conditions. The dominatee nodes of 7 include (4, 2, 5, 6, 1, 9). Nodes 5 and 6 are leaf nodes, so they remain as leaf nodes. Nodes 4 and 7 are not leaf nodes; moreover, they have only leaf neighbours, so one of the two nodes is included with the dominator node. Hence, Node 4 is included in the dominator and also in the CDS. The dominatee Nodes 1 and 9 have no further leaf neighbours, so these two nodes are not included in the CDS.

**Figure 2:**
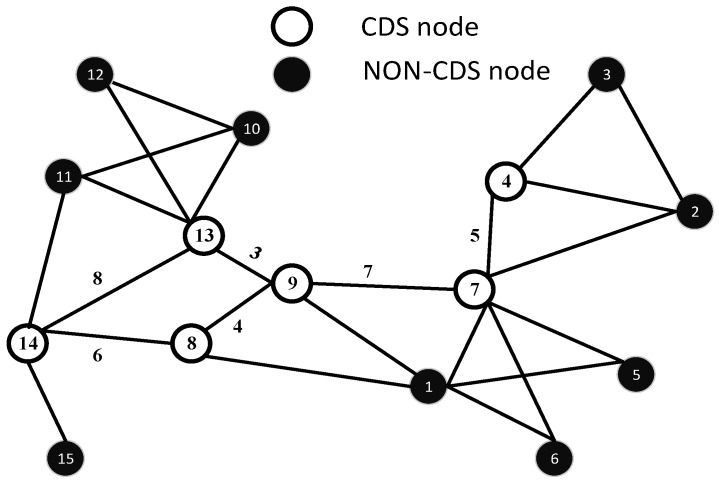
Formation of the CDS.

Now, consider the second highest degree node, which is Node 13. Node 13 is included in the dominator, as well as in the CDS. The dominatee nodes of 13 include (10, 12, 11, 14, 9). Nodes 10 and 12 are leaf nodes, and hence, these two nodes remain as dominatees. Node 14 is included in the dominator and the CDS, because it has a leaf neighbour, which is Node 15. Moreover, as we have mentioned, the node that is the dominatee of two different dominators is also included in the CDS. After applying all of the above-mentioned steps on the network, we get the set of CDS nodes, which includes (4, 7, 9, 13, 14, 8), as shown in Figure 2.

Definition 2: A undirected connected graph G=(V,E), such that a sub-graph $S^{\prime }$ of G is called the spanning tree that connects all of the nodes in V. An MST $S{}^{\prime \prime }$ is a sub-graph of $S^{\prime }$, such that the weight of $S{}^{\prime \prime }$ is less than or equal to the weight of $S^{\prime }$ of G. The weight of the spanning tree depends on the Euclidean length of the edge, $e\left(i,j\right)$ .

Definition 3: Given a undirected connected graph G=(V,E) with the formation of CDS and MST, an HC is defined as the path followed by the AUV that is accomplished through the CDS-MST, such that each edge is visited by the AUV once and returns to the starting node.

Definition 4: A CDS, V${}_{CDS}$⊂*V* of *G*, such that HC is transformed to a sub-optimized elliptical path of AUV. Moreover, V${}_{CDS}$⊂ V, such that the major and minor axis of the elliptical path *a* and *b* are sub-optimized to achieve: Maximize$\;d_{total}$*=*$\sum_{i=1}^{T_{GN}}(kr_{i}\;$*+*$d_{i}$*)*.

### 3.2. Trajectories of AUV Mobility

**Figure 3:**
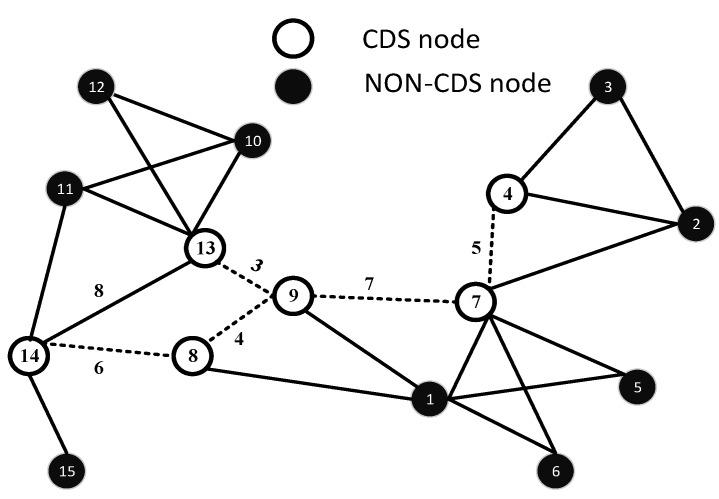
Formation of the CDS-MST on the basis of the Euclidean distance.

After the formation of the CDS, the MST is executed on the CDS as the baseline to formulate the HC, which is the trajectory of the AUV. The MST of the given CDS having links $\left\{l\left(4,7\right),l\left(7,9\right),l\left(9,13\right),l\left(8,9\right),l\left(8,14\right)\right\}$ is shown in Figure 3. *V* is further divided into V${}_{CDS}$ and V${}_{non-CDS}$ sets, such that V${}_{CDS}$⊂V and V${}_{non-CDS}$⊂V. We can define V of graph G as V = V${}_{CDS}$∪ V${}_{non-CDS}$, where V${}_{CDS}$ is a set of CDS nodes and V${}_{non-CDS}$ is a set of non-CDS nodes; moreover, V${}_{CDS}$∩ V${}_{non-CDS}$ = *∅*. Similarly, edges, E, are subdivided into E${}_{CDS}$ and E${}_{non-CDS}$, such that, E = E${}_{CDS}$∪ E${}_{non-CDS}$. E${}_{CDS}$ is a set of edges between two CDS nodes. E${}_{non-CDS}$ is a set of edges between non-CDS nodes or CDS and non-CDS nodes. Edges in E${}_{CDS}$ are further divided into E${}_{MST}$ and E${}_{non-MST}$. According to Definition 1 and Definition 2, all vertices of V${}_{CDS}$ are connected with each other through edges, E${}_{CDS}$. The path for the AUV is accomplished along E${}_{MST}$, which is the sub-optimal random track.

After establishment of the CDS, the criterion for the traversing of the AUV is as follows. There are three lists that are maintained to find the next hop of the AUV:

- Forward traversed list (FTL)
- Reverse traversed list (RTL)
- Trajectory list (TL)

An MST is constructed by initiating from the highest ID of the CDS and by adding edges in the MST, which have less weights. FTL is always scanned first to find the shortest outgoing edge. RTL is scanned, if FTL is empty. Once the leaf node of the CDS reached, the incoming edge is immediately traversed back. When all of the links are added to the TL and the current node is a starting node, then the HC is successfully constructed.

#### 3.2.1. Formation of the HC: An Example of AUV Trajectory Formation

Let random weights be assigned in terms of the Euclidean distance for each edge e ∈*E* of *G*. Let the Euclidean distance between nodes be represented by *d*; then, D${}_{ij}$ = {d(8,9) = 4, d(7,9) = 7, d(4,7) = 5, d(9,13) = 3, d(13,14) = 8, d(8,14) = 6}, as shown in Figure 2. Initially FTL, RTL and TL have the following status: FTL = $\left\{l\left(4,7\right),l\left(7,9\right),l\left(9,13\right),l\left(8,9\right),l\left(8,14\right)\right\}$, RTL = *∅* and TL = *∅*. We start from the node with the highest ID in V${}_{CDS}$. In our case, the highest ID node is 14, e(14,8), which is added to TL = $\left\{e\left(14,8\right)\right\}$ and $l\left(8,14\right)$ is moved to the RTL. At Node 8, e(8,9) is added to TL = $\left\{e\left(14,8\right),e\left(8,9\right)\right\}$, and similarly, $l\left(9,8\right)$ is moved to the RTL. At Node 9, we have two choices $e\left(9,13\right)$ and $e\left(9,7\right).$ The Euclidean length of $e\left(9,13\right)$ is shorter than that of $e\left(9,7\right)$, so $e\left(9,13\right)$ is included in TL = $\left\{e\left(14,8\right),e\left(8,9\right),e\left(9,13\right)\right\}$, and $e\left(13,9\right)$ is added to the RTL. At Node 13, there is no more outgoing edge of CDS, so $e\left(13,9\right)$ is added to TL = $\left\{e\left(14,8\right),e\left(8,9\right),e\left(9,13\right),e\left(13,9\right)\right\}$. At Node 9, we have two options, one from the FTL and the other from the RTL; $e\left(9,8\right)$ is in the RTL, and $e\left(9,7\right)$ is in the FOL . $e\left(9,7\right)$ is chosen because it lies in the FTL. Therefore, TL = $\left\{e\left(14,8\right),e\left(8,9\right),e\left(9,13\right),e\left(13,9\right),e\left(9,7\right)\right\}$. At Node 7, we have only one option, so $e\left(7,4\right)$ is added in the TL, and $e\left(4,7\right)$ is added to the RTL. Node 4 is a leaf node and, hence, traversed back, and $e\left(4,7\right)$ is added to the TL. At Node 7, we have one option, so $e\left(7,9\right)$ is added to the TL. At Node 9, we have two options: either go to 13 or 8. As $e\left(9,13\right)$ is already added in the TL, so HOL is scanned, and the next destination is Node 8. Similarly, the TL is constructed by continuing on this line, and we have,

(1) $$TL=\left\{\begin{array}{c} e\left(14,8\right),e\left(8,9\right),e\left(9,13\right),e\left(13,9\right),e\left(9,7\right),e\left(7,4\right),e\left(4,7\right),\\ e\left(7,9\right),e\left(9,8\right),e\left(8,14\right), \end{array}\right\}$$

**Figure 4:**
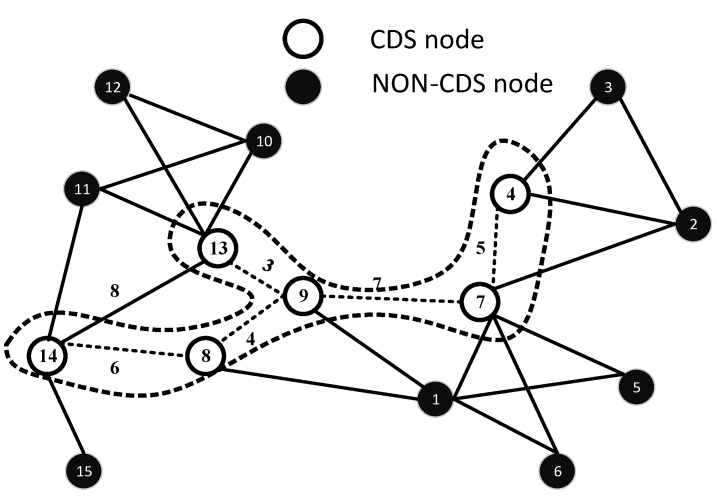
Trajectory of the AUV along the HC.

The length of the AUV path can be computed easily by adding all links of the CDS-MST as follows. The HC is shown in Figure 4.

(2) $$Lp=\sum_{\forall \;e(i,j)\in EMST}length(i,j)+\alpha$$

where length(i,j)∈EMST is the distance between node *i* and *j*. *α* is an offset factor, if the trajectory deviates from the CDS nodes. *α* is used to compensate the distance margin. length(i,j) is computed by using the Euclidean distance formula:

(3) $$d\left(i,j\right)=\sqrt{{\left(x_{i}-x_{j}\right)}^{2}+{\left(y_{i}-y_{j}\right)}^{2}}$$

where $d\left(i,j\right)$ is the Euclidean distance between *i* and *j*.

#### 3.2.2. Circular Trajectory

The irregular trajectory of the AUV along the HC can be changed to a circular trajectory as follows. Since we know that the area of a circle:

(4) $$A=\pi r^{2}$$

where *r* is the radius of the circle. To compute *r*, we have: the circumference of a circle is C=2πr, where:

(5) $$r=\;\frac{C}{2\pi \;}$$

(6) $$C=Lp=2\times (\sum_{\forall \;e(i,j)\in EMST}d\left(i,j\right)+\alpha )$$

The detailed steps to find the area of the circular trajectory are given in Appendix A.

(7) $$A=\frac{({\sum_{\forall \;e(i,j)\in EMST}d\left(i,j\right)+\alpha )}^{2}}{\pi }$$

Firstly, the MST is established, and then, the random path (MST) is converted into a circular path. After the adjustment of some parameters, it is converted into an elliptical path. As we know that the circumference of a circle is equal to the length of a random path (*i.e.*, MST), that is why C=Lp. Later on, it will become clear that this setting depends on the value of *β*, which is sub-optimized via Monte Carlo simulation. In order to match the changing GNs with the fixed route of the AUV, the GNs are selected within the communication range of the AUV, and a synchronization mechanism is defined between these.

#### 3.2.3. Elliptical Trajectory

The path of the AUV plays a vital role in efficient data gathering. A number of trajectories, like linear trajectory, circular trajectory, square-shaped trajectory and random way-points trajectory, exist in the literature. However, these trajectories have three major drawbacks: (i) high end-to-end delay; (ii) low packet delivery ratio; and (iii) high energy consumption. We, therefore, focus on the sub-optimized elliptical trajectory of the AUV, rather than the actual setting of the nodes for the network lifetime prolongation, throughput maximization and end-to-end delay minimization. For example, in the case of the MST, there is no uniform movement of the AUV, which results in data loss (the packet dropped ratio increase). Nodes lying in the corner of the network area are at farther distances from the MST and, thus, need surplus energy to transmit data packets to the GNs. This type of movement of the AUV on the MST leads to non-uniform consumption of energy. Therefore, we convert the random trajectory (*i.e.*, the actual setting of the nodes or the MST) into an elliptical trajectory to minimize the non-uniform consumption of energy. Moreover, the pattern of the GNs is also disturbed due to the random path followed by the AUV on the MST.

**Figure 5:**
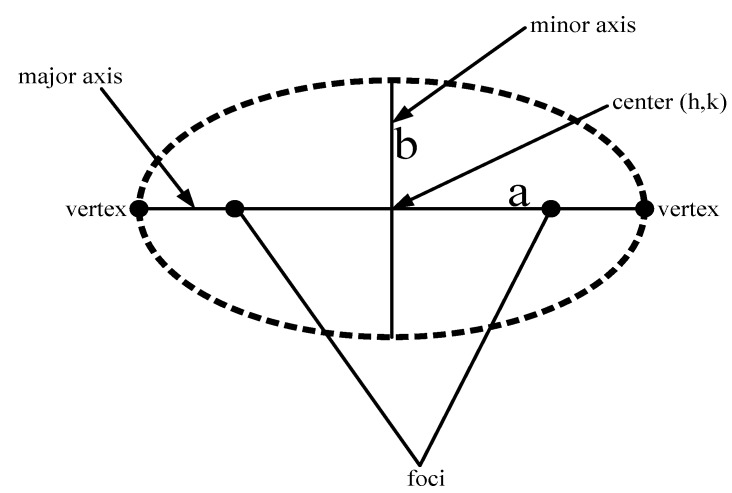
Elliptical trajectory of the AUV.

The major and minor axis of the ellipse are *2a* and *2b*, respectively, as shown in Figure 5. The major and minor axis are important factors to calculate the area of an ellipse. In other words, we can say that if the optimal area of an ellipse is calculated, then the network throughput is increased. If the area of the first quadrant is determined, then it is easier to compute the area of the remaining three quadrants. Therefore, we focus on finding the area of ellipse ($A_{ellipse}$) by using the general equation as follows:

(8) $$\frac{x^{2}}{a^{2}}+\frac{y^{2}}{b^{2}}=1$$

The detailed steps to compute the area of the first quadrant of ellipse ($A_{FQ-ellipse}$) is given in Appendix B. After further simplification, we have calculated:

(9) $$A_{FQ-ellipse}=\frac{ab\pi }{4}$$

Let b=r; then, we can write:

(10) $$b=\frac{\sum_{\forall \;e(i,j)\in EMST}d\left(i,j\right)+\alpha }{\pi }$$

The relation between the semi-major and semi-minor axes in terms of *β* is given. The sub-optimized value of *β* is calculated in the next section.

(11) a=b+bβ0<β,<1,a>b

**Figure 6:**
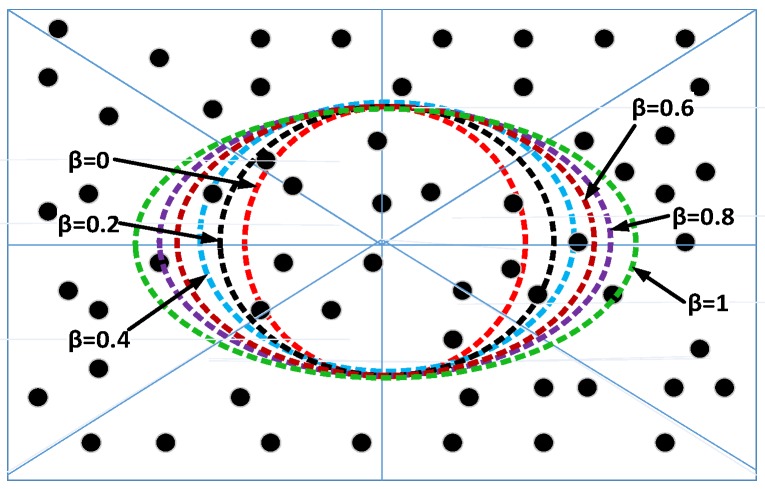
Different values of *β* for different trajectories of the AUV.

**Figure 7:**
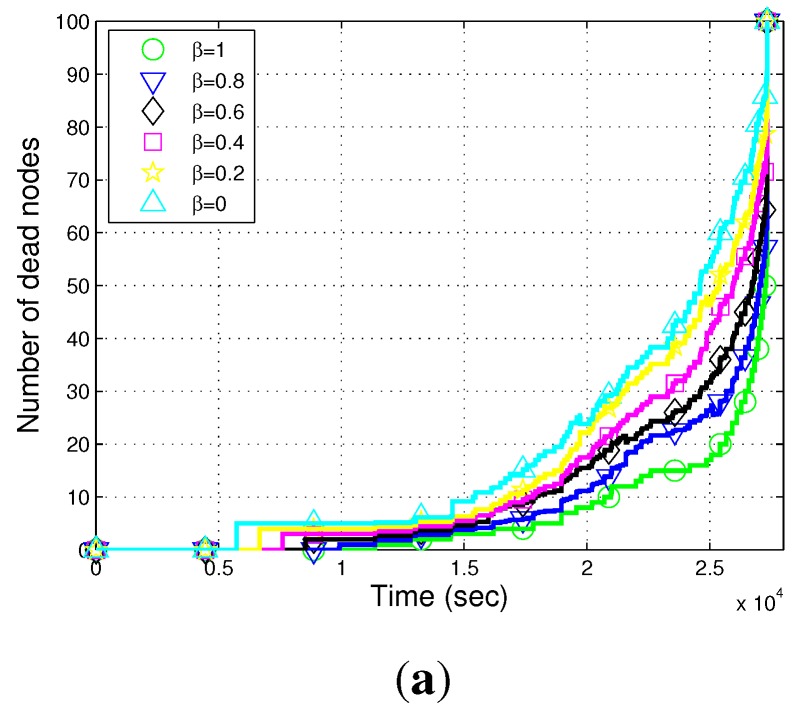

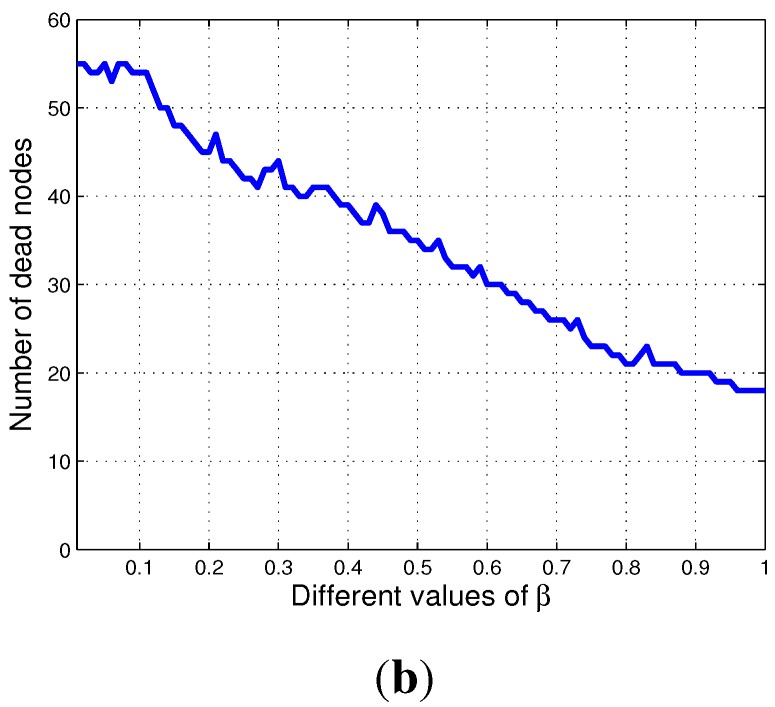
Impact of *β*. (**a**) The number of dead nodes of the AEDG for different values of *β*; (**b**) the number of dead nodes of the AEDG for different values of *β* at a specific time instant.

Figure 6 shows that we have simulated different trajectories of the AUV by varying the value of *β*. When the value of *β* is equal to zero, the trajectory of the AUV is circular. In the circular trajectory, the distance between end nodes and the AUV increases, and hence, the number of transmission(s) and reception(s) also increases. This leads to increased energy consumption in transmitting and receiving the sensed data. As we increase the value of *β* from zero, the distance between end nodes and the AUV decreases, which leads to a decreased number of hops. A reduced number of hops results in low transmissions and receptions, which leads to a decrease in the energy consumption. Therefore, the maximum number of nodes are alive for a longer duration by increasing the value of *β*, as shown in Figure 7a,b. Simulation results with varying values of *β* depict that the majority of the nodes are alive for a longer duration when the value of *β* is equal to one. Hence, the best results for β=1 prove the consideration to prefer the elliptical trajectory.

If the nodes are located on a straight line, then going on the straight line along the location of nodes seems to be more logical. However, as per our considerations, the network area is a 3D cube in which nodes are randomly deployed. If the AUV moves as per the actual settings of nodes, then random movements at surplus positions would lead to very high end-to-end delay. On the other hand, we need to gather maximum data from all parts of the network area. In this regard, an elliptical trajectory provides relatively enhanced results as compared to other possible AUV trajectories, like circular, cubic, rectangular and square.

## 4. Sub-Optimal Data Gathering

According to Definition 4, the values of *a* and *b* are sub-optimized to form a sub-optimal elliptical path of the AUV in order to maximize the total amount of data. As we have two types of nodes/vertices, such as V${}_{CDS}$ and V${}_{non-CDS}$., V${}_{CDS}$ nodes and E${}_{CDS}$ edges form the elliptical path of the AUV, whereas, V${}_{non-CDS}$ node and E${}_{non-CDS}$ edges are used for data forwarding to the GN. In this case, we divide the total number of hops of all of the network into two categories:

- CDS hops (h${}_{CDS}$)
- Non-CDS hops (h${}_{non-CDS}$)

In other words, we can say that CDS hops are equal to $h{}_{CDS}=N\left(E{}_{CDS}\right)$, and non-CDS hops are equal to $h{}_{non-CDS}=N\left(E{}_{non-CDS}\right)$, where *N* represent total number of CDS edges or non-CDS edges.

Therefore, according to Definition 4, our objective function to maximize $\;d_{total}$ = $\sum_{i=1}^{T_{GN}}(kr_{i}\;$+ $d_{i}$) for efficient data gathering is transformed into minimizing $\sum_{i=1}^{non-CDS}hi\;$, which ultimately depends on the elliptical trajectory of the AUV and also on the values of *a* and *b*.

Minimize:

(12) $$\sum_{i=1}^{non-CDS}h_{i}\;$$

Subject to:

(13) $$\sum_{j=1}^{CDS}h_{j}\;+\sum_{k=1}^{non-CDS}h_{k}\;\leq \sum_{i=1}^{n}h_{i}\;$$

In other words, we can say that in order to minimize $\sum_{i=1}^{non-CDS}h_{i}\;$, we have to maximize the $\sum_{i=1}^{CDS}h_{i}\;$.

As we have discussed earlier, that random or elliptical movement of the AUV is basically a combination of all traversed links of the CDS-MST or the number of hops in the CDS-MST. Therefore, the elliptical movement of the AUV is approximately equal to the number of CDS hops traversed by the AUV as given:

(14) P=2×NumberofCDS-hops

(15) $$P≅2\times \sum_{i=1}^{CDS}h_{i}\;$$

where *P* is the perimeter of the elliptical path traversed by the AUV. In other words, we can say that the perimeter is sub-optimized to maximize the total number of CDS hopes, which leads to minimized non-CDS hops. When the number of hops between the AUV trajectory and the end node is minimized, the multiple transmission and receptions are avoided in the harsh underwater environment. This leads to efficient data gathering with minimum energy consumption. According to Equation (15), the CDS hops depend on the perimeter, *P*, which depends on the eccentricity (*e*) of the ellipse, as well as the values of *a* and *b*. Therefore, the sub-optimal range of *a*, *b* and *e* leads to the sub-optimized perimeter or elliptical path of the AUV, which further leads to efficient data gathering.

Starting from the equation of the perimeter and solving for $h_{i}$, we reach the following equation (see Appendix C for details).

(16) $$\begin{array}{cc} \sum_{i=1}^{CDS-hops}h_{i} & =\left(\frac{\sum_{\forall \;e\left(i,j\right)\in EMST}d\left(i,j\right)+\alpha }{\pi }+\frac{\sum_{\forall \;e\left(i,j\right)\in EMST}d\left(i,j\right)+\alpha }{\pi }\;\beta \;\right)\\ & \times \pi [1-\sum_{i=1}^{\infty }\frac{{\left(2i\right)!}^{2}}{{\left(2^{i}.i\right)!}^{4}}\;\frac{{\left(\frac{\sqrt{\beta^{2}+2\beta }}{\;(1+\beta )\;}\right)}^{2i}}{2i-1}]\; \end{array}$$

After further simplification, we have the following expression (the detailed steps are given in Appendix D).

(17) $$\sum_{i=1}^{CDS-hops}h_{i}=b\pi \left(1+\beta \right)[1-\sum_{i=1}^{\infty }\frac{{\left(2i\right)!}^{2}}{{\left(2^{i}.i\right)!}^{4}}\;\frac{{\left(\beta^{2}+2\beta \right)}^{i}}{\left(2i-1)(1+\beta \right)}]\;$$

(18) $$\sum_{i=1}^{CDS-hops}h_{i}=\left[\sum_{\forall \;e\left(i,j\right)\in EMST}d\left(i,j\right)+\alpha \right]\left(1+\beta \right)[1-\sum_{i=1}^{\infty }\frac{{\left(2i\right)!}^{2}}{{\left(2^{i}.i\right)!}^{4}}\;\frac{{\left(\beta^{2}+2\beta \right)}^{i}}{\left(2i-1)(1+\beta \right)}]\;$$

From the above equation, we may conclude that $\sum_{i=1}^{CDS-hops}h_{i}\;$ directly relates to the value of *β*. We simulate our proposed technique with different values of *β* in the next section. It is worth mentioning here that Equations (16)–(18) are part of the derivation of the mathematical expression that shows the relation between the major and minor axes of the ellipse. As we have discussed, the major and minor axes of the ellipse play a vital role in increasing/decreasing the network throughput, which is why we have derived the mathematical expression for the major and minor axes. From Equation (18), we have proved that the CDS-hops (*i.e.*, the trajectory of the AUV) are directly proportional to *β*, which in turn plays a vital role in the calculation of the major and minor axes (*a* and *b*). Thus, we have performed Monte Carlo simulations to obtain the sub-optimum value of *β*.

Note: The major and minor axes (*a* and *b*) depend on the CDS and the non-CDS nodes. Equations (14)–(18) show the connection of the MST with the elliptical route. Equation (18) is about the relationship of the CDS-hops or MST with eccentricity, as well as the semi-major axis *a*. Therefore, Equation (18) deals with the relationship between the MST and the elliptical route (the elliptical route depends on the value of *a* and *e*).

### 4.1. Calculation of the Sub-Optimized Value of β

We simulate our protocol for different values of *β* at a time equal to $2.5\times 10^{4}$ s. Figure 7b shows the variation of *β* from 0–1 with a margin of 0.01. Figure 7b depicts that with the increasing value of *β*, the number of dead nodes decreases. The reason is that when the value of *β* is zero, the trajectory of the AUV is circular, and hence, the distance between the end node and the AUV increases. This leads to increased energy consumption on transmission(s), reception(s) and processing of aggregated data. However, when the value of *β* is equal to one, the multiple transmissions and receptions decrease due to the reduced number of hops. Therefore, the maximum nodes are alive at β=1, as shown in Figure 7b, compared to the previous values. Moreover, we simulate the network throughput of the AEDG for different values of *β*, as shown in Figure 7a. As we have discussed above, the maximum number of nodes are alive for a longer duration at β=1. Therefore, the network throughput is also maximized at β=1.

Moreover, we simulate our proposed protocol for different values of *β*, as shown in Figure 6. We simulate different trajectories of the AUV by varying the value of *β*, as shown in Figure 6. We use Equation (C2) to calculate the different elliptical paths of the AUV. The value of *β* plays a significant role to increase the stability period, as well as the throughput of the network. In our case, the value of *β* is varying from 0–1, as shown in Table 1. When the value of *β* is equal to zero, the trajectory of the AUV is circular. In the circular trajectory, the distance between end nodes and the AUV increases, and hence, the number of transmission(s) and reception(s) also increases. This leads to increased energy consumption in transmitting, receiving and processing the sensed data. As we increase the value of *β* from zero, the distance between end nodes and the AUV decreases, which leads to the decrease of the number of hops. A reduced number of hops results in low transmissions and receptions, which lead to a decrease in the energy consumption. Therefore, the maximum number of nodes are alive for a longer duration by increasing the value of *β*, as shown in Figure 7a,b. Simulation results with varying values of *β* depict that the majority of the nodes are alive for a longer duration when the value of *β* is equal to one. Hence, the majority of nodes are alive for a longer duration, which increases the network throughput, as shown in Figure 7b.

**Table 1:** Different values of *β*.

*β*	b	a = b + b*β*
0	50 m	50 m
0.2	50 m	60 m
0.4	50 m	70 m
0.6	50 m	80 m
0.8	50 m	90 m
1	50 m	100 m

## 5. AEDG: The Proposed Scheme

**Figure 8:**
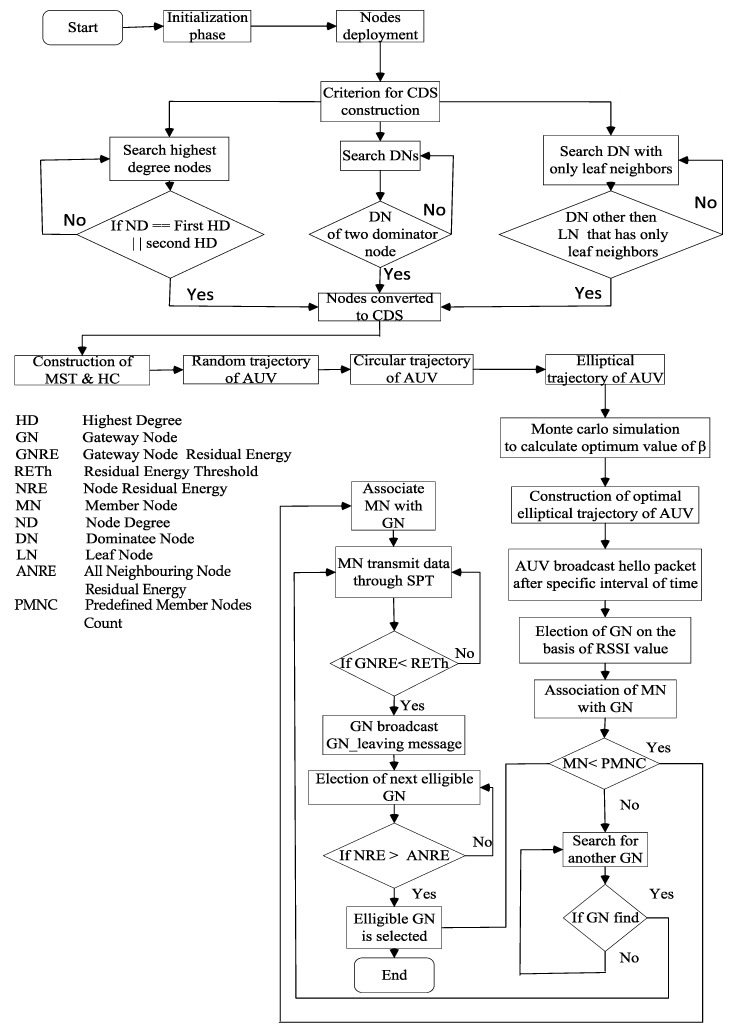
AEDG: protocol operation.

Besides the setup of the CDS-based elliptical route, the selection and rotation of GNs and the MST-based association of the MNs with the GNs, we also propose an efficient data gathering scheme to enhance the network throughput and to preserve the network’s energy. To prolong the network lifetime, the AEDG employs the AUV to collect the data from the GNs. To minimize the energy consumption, we use an SPT algorithm by associating the MNs with the GNs and devise a criterion to limit the association count of the nodes. Moreover, the role of the GNs is rotated to balance the energy consumption. We also develop the sub-optimal elliptical trajectory of the AUV by using the CDS to enhance the throughput of the network. The detailed flow chart of our proposed scheme is shown in Figure 8.

In our proposed protocol, the surface sink is deployed on the water surface. This surface sink has both radio and acoustic modems. Underwater sensor nodes are deployed at the bottom of the region. The AUV moves in an elliptical path in order to collect the data from the GNs. The following assumptions are taken for the proposed protocol.

- The AUV has unlimited power, memory and computational capability.
- The trajectory of the AUV is predefined (elliptical).
- The nodes are randomly deployed in the underwater environment.

### 5.1. Initialization Phase

In the initialization phase, the AUV moves in an elliptical path in order to select the GNs. The AUV periodically broadcasts hello packets. The flow chart in Figure 8 describes the initialization phase in more detail. The following subsection describes the initialization phase.

#### 5.1.1. GN Selection Criterion

**Figure 9:**
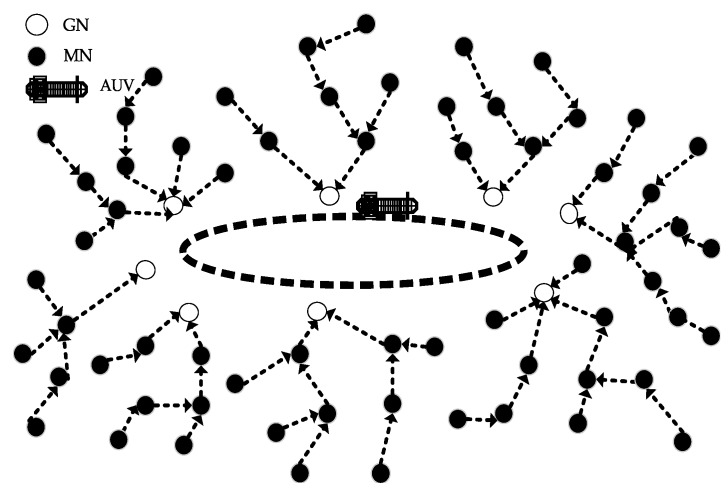
Association of member nodes with the GN through the SPT and communication between the GN and the AUV.

In the AEDG, an AUV moves along a fixed elliptical trajectory, as shown in Figure 9. The selection of the GN is based on the RSSI value and the residual energy of the individual node. The GN election is carried out multiple times in our proposed scheme, as shown in Figure 8. The criterion for the selection of the GN and the association of the member nodes is given below in detail.

The AUV regularly broadcasts hello packets after a specific interval of time. Nodes within direct communication range of the AUV listen for the hello packet and broadcast it to their neighbours. Each node calculates the distance between itself and the AUV. The node whose RSSI value is greater is selected as the GN. As the network evolves, the energy of the GN is consumed with time, so in order to avoid the “hot spot problem”, the GN is rotated when its energy drops below a pre-defined threshold.

Moreover, the GNs are selected from the region in direct communication range of the AUV. In our protocol, the communication range of the AUV is greater than that of the AEERP. The stability period of the AEDG is greater than the AEERP, because the area for the selection of the GNs increases due to the long communication range of the AUV as compared to the AEERP, and hence, more nodes are eligible for GN selection. Moreover, the restriction on the number of member nodes with the GN enhances the stability period and reduces the burden on the GNs.

#### 5.1.2. Member Nodes’ Association

After the selection of the GN, the member nodes join the GN through the SPT algorithm. In the SPT, nodes forward the data from the downstream nodes to the upstream nodes. In the selection of the SPT, every node shares its RSSI value with its neighbours. The member node with the highest RSSI value is selected as the next hop. The member nodes transmit their data while finding the route via the SPT algorithm. The SPT is based on the RSSI value of the hello packet that is received by each member node from the AUV. After making the SPT, the member nodes transmit their data to the GN in a multi-hop fashion. Finally, the GN forwards the aggregated data to the AUV by using a short-range acoustic link. The sub-optimal assignment of member nodes with the sub-optimal GN is describe in the flow chart of Figure 8.

### 5.2. Data Collection Phase

In the data transmission phase, nodes first sense data and then forward them to the next node by using the SPT. Finally, the data are received at the GN, which transmits them to the passing AUV. As a large amount of data is relayed by the GN, it depletes its energy more quickly, thereby reducing the network lifetime. In order to increase the lifetime of the network, the idea of rotating the GNs is introduced. Each node that is within direct communication range of the AUV is eligible to become the GN if its residual energy is greater than the rest of the nodes. The GN periodically checks the residual energy after a specific interval. If the residual energy reaches its threshold, the GN broadcasts the GN leaving message. After listening to this message, the nodes share their information with each other, and the node with the highest residual energy is selected as the GN. As the network evolves, the GNs are rotated, and the member nodes attached associate with their respective the GNs by using the SPT algorithm. Hence, in the next cycles, the AUV communicates with the next selected GN. Figure 9 shows the formation of the SPT and the data transmission of the member nodes to the GNs. The member nodes sense, as well as forward the aggregated data to the next node, and then, the data are destined for the GNs. Finally, these GNs transmit the aggregated data to the passing AUV.

## 6. Simulation Results

We validate and evaluate our proposed scheme via simulations, where our proposed AEDG protocol is compared to two existing protocols: AURP and AEERP. We vary the network area between 100 m × 100 m and 10 km × 10 km, where 10 and 100 nodes and one AUV are deployed. The nodes are initially equipped with 100 J. We assume a packet size of 70 bytes. Each sensor node has a fixed transmission range of 20 m. The LinkQuest UWM1000 [20] acoustic modem is used, having a 10-kbps bit rate. The AUV moves on the sub-optimized elliptical path. Simulation parameters are given in Table 2. We use the following metrics for the performance evaluation.

**Table 2:** Parameters used in the simulation.

Parameters	Values
Network size	From 100 m × 100 m to 10 km × 10 km
Number of nodes	10 and 100
Initial energy of normal nodes	100 J
Data aggregation factor	0.6
Packet size	70 bytes
Transmission range of the sensor node	20 meters
Number of AUVs	1

### 6.1. Network Performance Parameters: Definitions

#### 6.1.1. Stability Period

The time from the start of the network till the death of the first node is called the stability period. It is measured in seconds.

#### 6.1.2. Network Lifetime

The time duration from network initialization till the death of the last node is measured in seconds.

#### 6.1.3. Throughput

This is defined as the number of successfully received data packets at the sink. Throughput is measured in packets/second.

#### 6.1.4. End-to-End Delay

End-to-end delay is defined as the time taken by the data packet to reach from the source to the destination. It is measured in seconds.

#### 6.1.5. Transmission Loss

This is the average signal loss between intermediate nodes during data forwarding. It is measured in decibels (dBs).

### 6.2. Network Performance Parameters: Discussions

This subsection discusses the network parameters that are used to evaluate the performance of our proposed scheme in comparison to the selected existing schemes.

#### 6.2.1. Network Lifetime

**Figure 10:**
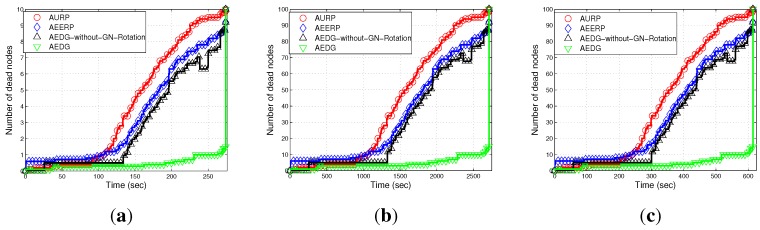
Number of dead nodes. (**a**) 10 nodes deployed in 100 m × 100 m; (**b**) 100 nodes deployed in 100 m × 100 m; (**c**) 100 nodes deployed in 10 km × 10 km.

Figure 10a–c shows the stability period of AURP, AEERP, AEDG and AEDG without GN rotation. In AURP, the GN is selected on the basis of the minimum distance and is equipped with extra energy. As the network evolves, the GN is rotated on the basis of the minimum distance. The stability period of the AURP decreases due to the unbalanced energy consumption. The next GN is selected when the first one dies, which leads to the decreased stability period. In AEERP, the GNs are selected on the basis of the RSSI value of hello packets transmitted by the AUV. As the network evolves, the nodes that exist in the direct communication range of the AUV are selected as the GNs on the basis of residual energy. The residual energy threshold is defined to balance the energy consumption. In this way, the energy consumption is balanced throughout the network lifetime. Hence, the AEERP has a greater stability period compared to the AURP. However, if a large number of member nodes are associated with the GN, its energy is quickly depleted, which leads to the creation of an energy hole (the rest of the network is disconnected). In AURP, the GNs, which are extensively involved in the data relaying, are fixed (not rotated over time). Thus, their batteries are drained earlier than the other nodes, as is evident from the jump in Figure 10a–c. In AEDG, the sub-optimal number of member nodes are associated with the GNs. In other words, the member nodes associated with the GNs are limited in number, which causes less energy consumption of the GN, and hence, the maximum number of nodes are alive for a long duration. Moreover, the residual energy threshold mechanism balances the energy consumption, which ultimately increases the stability period, and the effect of the GNs rotation is also clearly visible.

#### 6.2.2. Path Loss

**Figure 11:**
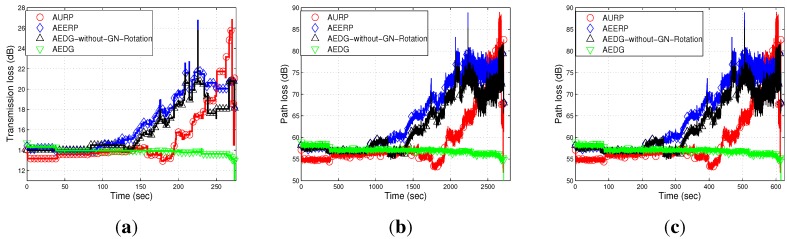
Path loss. (**a**) 10 nodes deployed in 100 m × 100 m; (**b**) 100 nodes deployed in 100 m × 100 m; (**c**) 100 nodes deployed in 10 km × 10 km.

Path loss depends on the distance between the sender and receiver and is affected by wave movement. Path loss is calculated by using the MMPE model. Figure 11a–c depicts the comparison of the path loss of AURP, AEERP, AEDG and AEDG without the GN rotation schemes in three different scenarios. In AURP, the intermediate nodes die out more quickly, and hence, the path loss increases. As there is no restriction on the association of member nodes, so the energy of the GNs quickly depletes. As nodes start dying, the distance between the sender and receiver increases; thus, the path loss increases. In AEDG, due to the balanced energy consumption, the path loss almost remains constant throughout the network lifetime and increases abruptly at the end due to the long haul communication of the far end nodes. Figure 11a,b shows the impact of node density on path loss; a high node density means a relatively greater number of communication links (distant communication), which leads to high path loss. On the other hand, Figure 11c shows the impact of network area on path loss, *i.e.*, direct relation. However, the impact of the number of links is far greater than the impact of the network area, as depicted in these figures.

#### 6.2.3. End-to-End Delay

**Figure 12:**
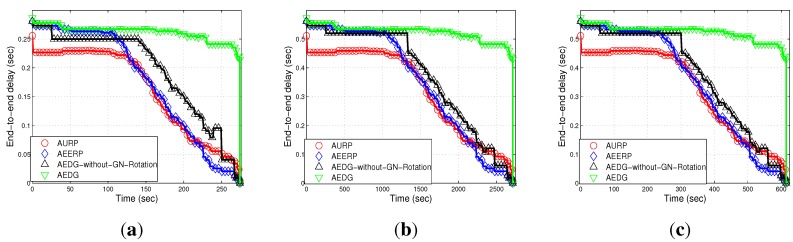
End-to-end delay. (**a**) 10 nodes deployed in 100 m × 100 m; (**b**) 100 nodes deployed in 100 m × 100 m; (**c**) 100 nodes deployed in 10 km × 10 km.

Figure 12a–c shows the end-to-end delay of the compared schemes (*i.e.*, AURP, AEERP, AEDG and AEDG without GN rotation) subject to node density and network area increase. In AEDG, nodes remain alive for a long duration and transmit data packets through the SPT. End-to-end delay depends on the distance between communicating nodes and the speed of the acoustic signal. As the speed of the acoustic signal is almost constant (1450 m/s), end-to-end delay only depends on the transmission distance. In AEDG, the maximum number of nodes are alive for a longer duration, and each node transmits over a certain distance. Therefore, the end-to-end delay of the AEDG is greater than the AURP and the AEERP. In our protocol, there is a trade-off between end-to-end delay and network throughput. Figure 12a–b shows the effect that node density has on end-to-end delay; a high node density means a relatively greater number of communication links (distant communication), which leads to a high end-to-end delay. On the other hand, Figure 11c shows the direct impact of the network area on the end-to-end delay. Moreover, these figures show that the impact of the node density is far greater than the impact of the network area.

#### 6.2.4. Network Throughput

**Figure 13:**
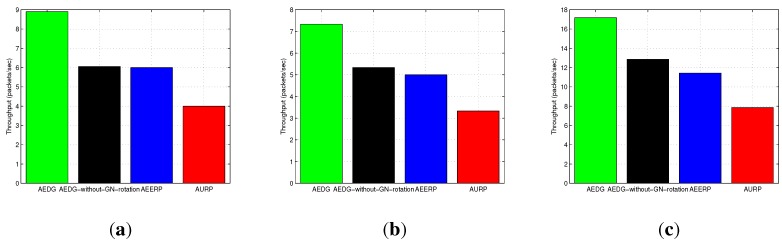
Network throughput. (**a**) 10 nodes deployed in 100 m × 100 m; (**b**) 100 nodes deployed in 100 m × 100 m; (**c**) 100 nodes deployed in 10 km × 10 km.

**Figure 14:**
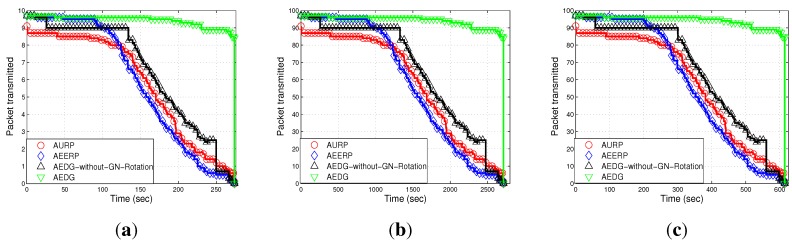
Packet transmission rate. (**a**) 10 nodes deployed in 100 m × 100 m; (**b**) 100 nodes deployed in 100 m × 100 m; (**c**) 100 nodes deployed in 10 km × 10 km.

Figure 13a–c shows the throughput of AURP, AEERP, AEDG and AEDG without GN rotation in different node densities and different network areas. In AEDG, the maximum number of nodes is alive for a long duration, as the restriction on the GNs enhances the stability period, and more nodes are available to relay the data of far end nodes; thus, Figure 14a–c shows that the packet transmission rate of the AEDG is better than the AURP and the AEERP, respectively. The AEDG has enhanced network throughput as compared to the AURP and the AEERP, because nodes transmit packets for a longer duration. In this way, the chances of packet dropping decrease, and throughput increases. Figure 13a–c and Figure 14a–c show the impact that node density and network area have on throughput and the packet transmission rate, respectively; a high node density means a relatively high packet transmission rate, which ultimately leads to a relatively high network throughput. Moreover, these figures clearly illustrate the impact of the GNs’ rotation.

#### 6.2.5. Transmission Loss

**Figure 15:**
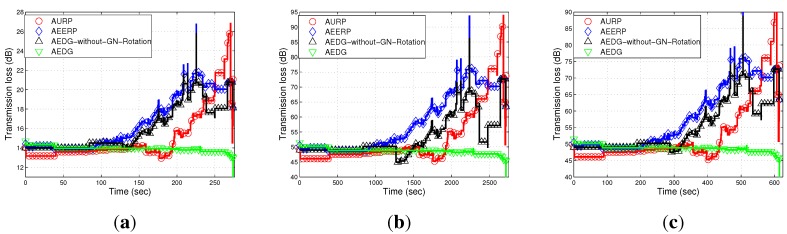
Transmission loss. (**a**) 10 nodes deployed in 100 m × 100 m; (**b**) 100 nodes deployed in 100 m × 100 m; (**c**) 100 nodes deployed in 10 km × 10 km.

The transmission loss of the compared schemes, which is computed by using the Thorp model, is shown in Figure 15a–c. Transmission loss depends on the transmission distance, bandwidth efficiency and the attenuation loss of the transmitted signal. A larger distance between nodes causes a high transmission loss, which further increases, due to the death of intermediate nodes. In the case of AURP and AEERP, the intermediate nodes quickly die due to the excessive burden, which leads to relatively high transmission loss in these schemes. The distant nodes transmit data at a longer distance, which increases the transmission loss at the end of the network lifetime. The transmission loss of AEDG is less due to balanced energy consumption and the longer stability period. Figure 15a,b shows the impact of node density on the transmission loss; high node density means a relatively greater number of communication links, which leads to high transmission loss. On the other hand, Figure 15c shows the direct relation between network area and transmission loss. To sum up, these figures show that the relative impact of node density on transmission loss is far greater than the relative impact of the network area on transmission loss.

### 6.3. Performance Trade-Offs

In this section, we discuss the performance of our routing protocol in terms of achievements and drawbacks. The trade-offs of the selected existing protocols with our proposed scheme are listed in Table 3. The AURP lacks a residual energy threshold mechanism at the GN, which results in the unbalanced energy consumption mechanism. Figure 10a–c depicts the stability period and the existence of alive nodes in the network. The AURP achieves low end-to-end delay at the cost of high energy consumption. Moreover, extra burden on the intermediate nodes, due to the relaying of a huge amount of data, leads to the transmission loss increases at the end, as shown in Figure 15a–c.

**Table 3:** Performance trade-offs made by the routing protocols.

Protocols	Mechanism	Advantages Achieved	Price Paid/at the Cost of
AURP	No mechanism to balanced energy consumption (Figure 10).	Less end-to-end delay (Figure 12).	Low stability period, high energy consumption and high transmission loss (Figure 10).
AEERP	Mechanism to balance energy consumption (Figure 10) and no mechanism to limit the association of MNs with the GN.	Less end-to-end delay (Figure 12) and enhanced throughput as compared to AURP (Figure 13).	High energy consumption and low stability period as compared to AEDG (Figure 10).
AEDG	Sub-optimized elliptical path of the AUV and sub-optimal assignment of member nodes with the GN.	High stability period, low energy consumption (Figure 10), high network throughput (Figure 13) and low transmission loss (Figure 15).	High end-to-end delay (Figure 12).

The AEERP has a mechanism to balance the energy consumption. However, the excessive association of the member nodes with the GN decreases its stability period, as shown in Figure 10a–c. The AEERP achieves reasonably high throughput (*cf.* Figure 13a–c) due to the introduction of the GN residual energy threshold mechanism, as compared to the AURP. Moreover, the AEERP achieves less end-to-end delay (*cf.* Figure 12a–c) at the cost of high energy consumption (*cf.* Figure 10a–c) as compared to the AEDG. In AEDG, the sub-optimized value of *β* for the elliptical path of the AUV is calculated through the Monte Carlo simulation method in order to increase the throughput with reduced energy consumption, as depicted in Figure 7a,b. Moreover, the sub-optimal assignment of MNs with their respective GNs balances the energy consumption with the residual energy threshold mechanism. The AEDG achieves high throughput (*cf.* Figure 13a–c) at the cost of high end-to-end delay (*cf.* Figure 12a–c). Moreover, it achieves a high stability period (*cf.* Figure 10a–c) and low transmission loss (*cf.* Figure 15a–c) due to the balanced energy consumption at the cost of high end-to-end delay (*cf.* Figure 12a–c).

Note: If the GN is periodically rotated (time based) among the sensor nodes, then the energy is evenly consumed among nodes, such that pre-mature death among the nodes can be avoided; however, in consideration of the nodes’ residual energy causing unnecessary energy waste when the network becomes heterogeneous. On the other hand, if the GN is rotated on the basis of the residual energy, the then frequent GN rotation would consume surplus energy during the later simulation course. To better cope with this problem, the GN is rotated on the basis of the nodes’ periodic residual energy (the hybrid approach of the time-driven and residual energy-driven approaches). Thus, the GNs are rotated at the cost of signalling message (overhead) exchange between the AUV and the nodes to be selected as the GNs. However, this overhead has no significant impact on the energy efficiency and throughput degradation, as compared to the achievements made due to the GNs’ rotation. Since we have implemented periodic residual energy-based GN rotation, the GN rotation-based results do not show at the beginning an advantage compared to the non-rotating GN method (see Figure 10, Figure 11, Figure 12, Figure 13, Figure 14 and Figure 15).

## 7. Conclusions and Future Work

In this paper, we have presented an AUV-aided energy-efficient routing scheme for UWSNs. We have also presented a model for efficient data gathering and proposed a mobility model using CDS for the sub-optimal trajectory of the AUV. We addressed the problems of the low data delivery ratio, the energy hole and high energy consumption. We have calculated the sub-optimal value of *β* by using the Monte Carlo simulation method. Simulation results have proven that our protocol performs better than the AURP and the AEERP (in harsh oceanic condition(s)) in terms of data gathering and energy consumption. The throughput of the AEDG is 35% more than the AEERP and 41% more than the AURP. However, the end-to-end delay of the AEDG is 30% more than the AEERP and 38% more than the AURP.

In the near future, we will extend our work to a 3D network area with multiple AUVs having different trajectories. Moreover, we will exploit the physical layer to enhance the data rate in a harsh underwater environment.

## Appendix

### A. Area of the Circular Trajectory of the AUV

The area of the circular trajectory of the AUV is calculated by using the following detailed steps as given below.

(A1) $$r=\;\frac{C}{2\pi \;}$$

(A2) $$C=Lp=2(\sum_{\forall \;e(i,j)\in EMST}d\left(i,j\right)+\alpha )$$

(A3) $$r=\;\frac{2(\sum_{\forall \;e(i,j)\in EMST}d\left(i,j\right)+\alpha )}{2\pi \;}$$

(A4) $$r=\frac{\sum_{\forall \;e(i,j)\in EMST}d\left(i,j\right)+\alpha }{\pi }$$

(A5) $$A={\pi \;(\frac{\sum_{\forall \;e(i,j)\in EMST}d\left(i,j\right)+\alpha }{\pi })}^{2}$$

(A6) $$A=\frac{({\sum_{\forall \;e(i,j)\in EMST}d\left(i,j\right)+\alpha )}^{2}}{\pi }$$

### B. Area of the Elliptical Trajectory of the AUV

Let us start from the basic equation of an ellipse.

(B1) $$\frac{x^{2}}{a^{2}}+\frac{y^{2}}{b^{2}}=1$$

Simplify Equation (B1) in terms of y:

(B2) $$y^{2}=\frac{a^{2}b^{2}-x^{2}b^{2}}{a^{2}}$$

(B3) $$y=\pm \sqrt{\frac{a^{2}b^{2}-x^{2}b^{2}}{a^{2}}}$$

(B4) $$y=\pm \;\frac{b}{a}\sqrt{a^{2}-x^{2}\;}\;\;\;\;\;\;\;$$

We ignore the (-) sign, because we are in the first quadrant, so the equation becomes:

(B5) $$y=\;\frac{b}{a}\sqrt{a^{2}-x^{2}\;}\;\;\;\;\;\;\;$$

In order to find the area of the ellipse, we integrate it from zero to a.

(B6) $$\int_{0}^{a}\left(\frac{b}{a}\sqrt{a^{2}-x^{2}\;}\right)\mathrm{dx}$$

(B7) Let x=asinθthen

(B8) dx=acosθdθ

(B9) $$\frac{b}{a}\;\int_{0}^{\frac{\pi }{2}}\sqrt{a^{2}-{(a\sin\theta \;\;)}^{2}\;}\times a\cos\theta \;d\theta$$

(B10) $$\frac{b}{a}\int_{0}^{\frac{\pi }{2}}a\times a\sqrt{1-{(\sin\theta \;\;)}^{2}\;}\times \cos\theta \;d\theta$$

(B11) $$ab\;\int_{0}^{\frac{\pi }{2}}\sqrt{1-{(\sin\theta \;\;)}^{2}\;}\times \cos\theta \;d\theta$$

(B12) $$ab\;\int_{0}^{\frac{\pi }{2}}\sqrt{{(\cos\theta \;\;)}^{2}\;}\times \cos\theta \;d\theta$$

(B13) $$ab\;\int_{0}^{\frac{\pi }{2}}{(\cos\theta \;\;)}^{2}d\theta$$

(B14) $$ab\int_{0}^{\frac{\pi }{2}}\frac{(1+\cos2\theta \;\;)}{2}d\theta$$

After further simplification, we get:

(B15) $$A_{FQ-ellipse}=\frac{ab\pi }{4}$$

The area of the first quadrant of the ellipse = $\frac{ab\pi }{4}$. Let b=r; then, we can write:

(B16) $$b=\frac{\sum_{\forall \;e(i,j)\in EMST}d\left(i,j\right)+\alpha }{\pi }$$

(B17) $$a=\frac{\sum_{\forall \;e(i,j)\in EMST}d\left(i,j\right)+\alpha }{\pi }+\frac{\sum_{\forall \;e(i,j)\in EMST}d\left(i,j\right)+\alpha }{\pi }\beta$$

(B18) $$A_{ellipse}\;=\left(\frac{\left[\frac{\sum_{\forall \;e(i,j)\in EMST}d\left(i,j\right)+\alpha }{\pi }+\frac{\sum_{\forall \;e\left(i,j\right)\in EMST}d\left(i,j\right)+\alpha }{\pi }\;\beta \right]\;\frac{\sum_{\forall \;e(i,j)\in EMST}d\left(i,j\right)+\alpha }{\pi }\;}{4}\right)\pi$$

(B19) $$A_{ellipse}\;=\frac{1}{4}[{\left(\frac{\sum_{\forall \;e(i,j)\in EMST}d\left(i,j\right)+\alpha }{\pi }\right)}^{2}+\left({\frac{\sum_{\forall \;e(i,j)\in EMST}d\left(i,j\right)+\alpha }{\pi })}^{2}\beta \right]\pi$$

(B20) $$A_{ellipse}\;=\frac{{(\sum_{\forall \;e(i,j)\in EMST}d\left(i,j\right)+\alpha )}^{2}+{(\sum_{\forall \;e(i,j)\in EMST}d\left(i,j\right)+\alpha )}^{2}\beta }{4}$$

(B21) $$A_{ellipse}\;=\frac{{(\sum_{\forall \;e(i,j)\in EMST}d\left(i,j\right)+\alpha )}^{2}[1+\;\beta ]\;}{4}$$

The above equation is the area of the right top quadrant of the ellipse, so in order to find the area of the whole ellipse, multiply this equation by four. Finally, the area of the ellipse is computed as:

(B22) $$A_{ellipse}={(\sum_{\forall \;e(i,j)\in EMST}d\left(i,j\right)+\alpha )}^{2}[1+\;\beta ]$$

### C. Calculation of the Sub-Optimal Value of *e*, *a* and *b*

The values of *a* and *b* for the sub-optimal value of *e* lead to the sub-optimal *P* of the ellipse. Let the radius of the circle that we have calculated in Equation (A4) be equal to the minor axis of the ellipse as b=r; then, we can write:

(C1) $$b=\frac{\sum_{\forall \;e(i,j)\in EMST}d\left(i,j\right)+\alpha }{\pi }$$

The relation between b and a may be expressed as:

(C2) a=b+bβ0<β,<1,a>b

(C3) $$a=\frac{\sum_{\forall \;e(i,j)\in EMST}d\left(i,j\right)+\alpha }{\pi }+\frac{\sum_{\forall \;e(i,j)\in EMST}d\left(i,j\right)+\alpha }{\pi }\beta$$

By plugging the values of *a* and *b* into Equation (C3), we have,

(C4) $$e=\frac{\sqrt{{\left(\frac{\sum_{\forall \;e(i,j)\in EMST}d\left(i,j\right)+\alpha }{\pi }+\frac{\sum_{\forall \;e(i,j)\in EMST}d\left(i,j\right)+\alpha }{\pi }\;\beta \;\right)}^{2}-{\left(\frac{\sum_{\forall \;e(i,j)\in EMST}d\left(i,j\right)+\alpha }{\pi }\right)}^{2}}}{\frac{\sum_{\forall \;e(i,j)\in EMST}d\left(i,j\right)+\alpha }{\pi }+\frac{\sum_{\forall \;e(i,j)\in EMST}d\left(i,j\right)+\alpha }{\pi }\;\beta \;}$$

After further simplification, Equation (C4) may also be written as:

(C5) $$e=\frac{\sqrt{{\left(\frac{\sum_{\forall \;e(i,j)\in EMST}d\left(i,j\right)+\alpha }{\pi }+\frac{\sum_{\forall \;e(i,j)\in EMST}d\left(i,j\right)+\alpha }{\pi }\;\beta \;\right)}^{2}-{\left(\frac{\sum_{\forall \;e(i,j)\in EMST}d\left(i,j\right)+\alpha }{\pi }\right)}^{2}}}{\left(\frac{\sum_{\forall \;e(i,j)\in EMST}d\left(i,j\right)+\alpha }{\pi }+\frac{\sum_{\forall \;e(i,j)\in EMST}d\left(i,j\right)+\alpha }{\pi }\;\beta \;\right)}$$

(C6) $$e=\frac{\sqrt{{\left(\frac{\sum_{\forall \;e(i,j)\in EMST}d\left(i,j\right)+\alpha }{\pi }\;\beta \;\right)}^{2}+2\left(\frac{\sum_{\forall \;e(i,j)\in EMST}d\left(i,j\right)+\alpha }{\pi }\right)\left(\frac{\sum_{\forall \;e(i,j)\in EMST}d\left(i,j\right)+\alpha }{\pi }\;\beta \right)}}{(\frac{\sum_{\forall \;e(i,j)\in EMST}d\left(i,j\right)+\alpha }{\pi }+\frac{\sum_{\forall \;e(i,j)\in EMST}d\left(i,j\right)+\alpha }{\pi }\;\beta \;)\;\;}$$

(C7) $$e=\frac{\sqrt{{\left(\frac{\sum_{\forall \;e(i,j)\in EMST}d\left(i,j\right)+\alpha }{\pi }\;\right)}^{2}\beta^{2}+2{\left(\frac{\sum_{\forall \;e(i,j)\in EMST}d\left(i,j\right)+\alpha }{\pi }\;\right)}^{2}\beta }}{(\frac{\sum_{\forall \;e(i,j)\in EMST}d\left(i,j\right)+\alpha }{\pi }+\frac{\sum_{\forall \;e(i,j)\in EMST}d\left(i,j\right)+\alpha }{\pi }\;\beta \;)\;\;}$$

(C8) $$e=\frac{\sqrt{{\left(\frac{\sum_{\forall \;e(i,j)\in EMST}d\left(i,j\right)+\alpha }{\pi }\right)}^{2}\beta^{2}+2{\left(\frac{\sum_{\forall \;e(i,j)\in EMST}d\left(i,j\right)+\alpha }{\pi }\right)}^{2}\beta }}{\frac{\sum_{\forall \;e(i,j)\in EMST}d\left(i,j\right)+\alpha }{\pi }\left(1+\beta \right)\;}$$

(C9) $$e=\frac{\frac{\sum_{\forall \;e(i,j)\in EMST}d\left(i,j\right)+\alpha }{\pi }\sqrt{\beta^{2}+2\beta }}{\frac{\sum_{\forall \;e(i,j)\in EMST}d\left(i,j\right)+\alpha }{\pi }\left(1+\beta \right)\;}$$

(C10) $$e=\frac{\sqrt{\beta^{2}+2\beta }}{\;(1+\beta )\;},\;\;\;0<\beta <1$$

### D. CDS-Hops: Detailed Derivation Steps

By putting $\sum_{\forall \;e(i,j)\in EMST}d\left(i,j\right)+\alpha =b\pi$, in Equation (16), we have the following expression:

(D1) $$\sum_{i=1}^{CDS-hops}h_{i}=\left(\frac{b\pi }{\pi }+\frac{b\pi }{\pi }\;\beta \;\right)\pi [1-\sum_{i=1}^{\infty }\frac{{\left(2i\right)!}^{2}}{{\left(2^{i}.i\right)!}^{4}}\;\frac{{\left(\beta^{2}+2\beta \right)}^{i}}{\left(2i-1)(1+\beta \right)}]\;$$

(D2) $$\sum_{i=1}^{CDS-hops}h_{i}=\left[b\pi +b\pi \beta \right)[1-\sum_{i=1}^{\infty }\frac{{\left(2i\right)!}^{2}}{{\left(2^{i}.i\right)!}^{4}}\;\frac{{\left(\beta^{2}+2\beta \right)}^{i}}{\left(2i-1\right)\left(1+\beta \right)}]\;$$

(D3) $$\sum_{i=1}^{CDS-hops}h_{i}=b\pi \left(1+\beta \right)[1-\sum_{i=1}^{\infty }\frac{{\left(2i\right)!}^{2}}{{\left(2^{i}.i\right)!}^{4}}\;\frac{{\left(\beta^{2}+2\beta \right)}^{i}}{\left(2i-1)(1+\beta \right)}]\;$$

By replacing the value of *b* in Equation (D3), we have:

(D4) $$\sum_{i=1}^{CDS-hops}h_{i}=\left[\sum_{\forall \;e\left(i,j\right)\in EMST}d\left(i,j\right)+\alpha \right]\left(1+\beta \right)[1-\sum_{i=1}^{\infty }\frac{{\left(2i\right)!}^{2}}{{\left(2^{i}.i\right)!}^{4}}\;\frac{{\left(\beta^{2}+2\beta \right)}^{i}}{\left(2i-1)(1+\beta \right)}]\;$$

### E. List of Abbreviations

**Table E1:** List of acronyms.

UWSN	underwater wireless sensor network
MS	mobile sink
AUV	autonomous underwater vehicle
AEDG	AUV-aided efficient data gathering
SPT	shortest path tree
CDS	connected dominating set
GN	gateway node
MN	member node
RSSI	received signal strength indicator
AURS	autonomous underwater robotic system
RBCDRR	round-based clustering scheme for data redundancy resolving
ACOA	ant colony optimization algorithm
AFSA	artificial fish swarm algorithm
AURP	AUV-aided underwater routing protocol
AEERP	AUV-aided energy-efficient routing protocol
MST	minimum spanning tree
DS	dominating set
HC	Hamiltonian circuit
FTL	forward traversed list
RTL	reverse traversed list
TL	trajectory list

**Table E2:** List of symbols.

*G*	Graph
*V*	Set of vertices
*E*	Set of edges
$A_{DC}$	Direct communication area
$A_{MC}$	Multi-hop communication area
$A_{ellipse}$	Area of ellipse
$A_{FQ-ellipse}$	Area of the first quadrant of the ellipse
*n*	Number of sensor nodes
$D_{a}$	Acceptable delay
$V_{CDS}$	Set of CDS nodes
$V_{non-CDS}$	Set of non-CDS nodes
$E_{CDS}$	Set of edges between CDS nodes
$E_{non-CDS}$	Set of edges between non-CDS nodes
$E_{MST}$	A subset of $E_{CDS}$ that forms an MST
$E_{non-MST}$	A subset of $E_{CDS}$ that does not form an MST
*i* and *j*	Node indices
$S^{\prime }$	Spanning tree
$S^{\prime \prime }$	A sub-graph of $S^{\prime }$ that forms an MST
e(i,j)	Edge between *i* and *j*
*a*	Major axis of an ellipse
*b*	Minor axis of an ellipse
l(i,j)	Link between *i* and *j*
*d*	Euclidean distance
$T_{GN}$	Total number of GNs
*α*	Offset factor that is used to compensate the distance margin
$x_{i}$	x-coordinate of node *i*
$y_{i}$	y-coordinate of node *i*
*r*	Radius of a circle
*C*	Circumference of a circle
*A*	Area of a circle
$L_{p}$	Length of AUV path
$h_{CDS}$	CDS hops
$h_{non-CDS}$	Non-CDS hops
*N*	Total number (of CDS or non-CDS edges)
*P*	Perimeter of the elliptical path traversed by an AUV
*e*	Eccentricity of an ellipse
*β*	A control variable for different trajectories of an AUV
dB	Decibels
